# Chromatin condensation but not DNA integrity of pig sperm is greater in the sperm-rich fraction

**DOI:** 10.1186/s40104-023-00938-w

**Published:** 2023-11-06

**Authors:** Estel Viñolas-Vergés, Jordi Ribas-Maynou, Isabel Barranco, Camila Peres Rubio, Sergi Bonet, Jordi Roca, Marc Yeste

**Affiliations:** 1 Unit of Cell Biology, Department of Biology, Faculty of Sciences, University of Girona, Girona, Spain; 2 Biotechnology of Animal and Human Reproduction (TechnoSperm), Institute of Food and Agricultural Technology, University of Girona, Girona, Spain; 3 Department of Medicine and Animal Surgery, Faculty of Veterinary Science, University of Murcia, Murcia, Spain; 4 Catalan Institution for Research and Advanced Studies (ICREA), S08010 Barcelona, Spain

**Keywords:** Chromatin, Condensation, DNA integrity, Ejaculate fractions, Pig, Protamination, Sperm

## Abstract

**Background:**

Protamination and condensation of sperm chromatin as well as DNA integrity play an essential role during fertilization and embryo development. In some mammals, like pigs, ejaculates are emitted in three separate fractions: pre-sperm, sperm-rich (SRF) and post sperm-rich (PSRF). These fractions are known to vary in volume, sperm concentration and quality, as well as in the origin and composition of seminal plasma (SP), with differences being also observed within the SRF one. Yet, whether disparities in the DNA integrity and chromatin condensation and protamination of their sperm exist has not been interrogated.

**Results:**

This study determined chromatin protamination (Chromomycin A3 test, CMA_3_), condensation (Dibromobimane test, DBB), and DNA integrity (Comet assay) in the pig sperm contained in the first 10 mL of the SRF (SRF-P1), the remaining portion of the sperm-rich fraction (SRF-P2), and the post sperm-rich fraction (PSRF). While chromatin protamination was found to be similar between the different ejaculate fractions (*P* > 0.05), chromatin condensation was seen to be greater in SRF-P1 and SRF-P2 than in the PSRF (*P* = 0.018 and *P* = 0.004, respectively). Regarding DNA integrity, no differences between fractions were observed (*P* > 0.05). As the SRF-P1 has the highest sperm concentration and ejaculate fractions are known to differ in antioxidant composition, the oxidative stress index (OSi) in SP, calculated as total oxidant activity divided by total antioxidant capacity, was tested and confirmed to be higher in the SRF-P1 than in SRF-P2 and PSRF (0.42 ± 0.06 vs. 0.23 ± 0.09 and 0.08 ± 0.00, respectively; *P* < 0.01); this index, in addition, was observed to be correlated to the sperm concentration of each fraction (Rs = 0.973; *P* < 0.001).

**Conclusion:**

While sperm DNA integrity was not found to differ between ejaculate fractions, SRF-P1 and SRF-P2 were observed to exhibit greater chromatin condensation than the PSRF. This could be related to the OSi of each fraction.

**Supplementary Information:**

The online version contains supplementary material available at 10.1186/s40104-023-00938-w.

## Background

Reduced sperm motility and viability, high proportions of sperm with morphological abnormalities and an increased incidence of oxidative stress are known to be associated to impaired fertility [1, 2]. In addition, it has become increasingly apparent that intact DNA, and proper protamination and condensation of sperm chromatin are required for correct embryonic development and for maintaining pregnancy to term [3, 4]. Indeed, not only is sperm DNA fragmentation detrimental to fertility, but defects in sperm chromatin protamination and condensation have an adverse effect on fertilization and blastocyst development [5]. Oxidative stress, which reflects redox imbalance, is at present regarded as one of the causes of reduced sperm quality and altered chromatin integrity [6]. While motility analyses reveal differences between sperm [7–9], whether this is also the case for chromatin condensation, protamination and DNA integrity has not been investigated. Yet, understanding better the disparities between individual cells of the same ejaculate could allow selecting the best sperm population, also in terms of DNA integrity, chromatin protamination and condensation.

Semen consists of a mixture of sperm and a fluid composed of secretions coming from the epididymis and accessory sex glands, including bulbourethral glands, the prostate and seminal vesicles. Ejaculates from some mammalian species, like pigs, horses and even humans are expelled in different fractions, namely: pre-sperm fraction (with no sperm cells); sperm-rich fraction (SRF); and post-sperm rich fraction (PSRF), which differ in sperm concentration and in the origin and composition of seminal plasma (SP) [10]. In pigs, the SP of the pre-sperm fraction originates from urethral, bulbo-urethral and prostate glands. Besides, while the SP of the SRF is a mixture of epididymal, seminal vesicles and prostate secretions, that of the PSRF is composed of secretions from seminal vesicles, prostate and bulbo-urethral glands [10, 11]. Separating ejaculate fractions allows identifying the particularities of the sperm cells contained in each fraction and the different oxidation–reduction status resulting from the distinct amounts of antioxidants present in the fluid [12]. In humans, collecting split ejaculates is also aimed to identify the fraction with the highest sperm quality, despite not being of routine application in assisted reproduction [13]. Recent investigations showed that sperm from different fractions might differ in their quality and DNA integrity [14–16]. Although new systems to harvest split ejaculates were designed to investigate differences between fractions [17], collection of human samples has an elevated risk of bias, as it highly depends on the ability of patients to separate ejaculate portions. This inconvenient may be circumvented by involving animal models with a more standardized semen collection, like the pig [18, 19]. Because the production and conservation of semen doses for artificial insemination is a routine procedure in this species, ejaculate collection is highly standardized and provides the opportunity of obtaining separate fractions with high volume, opening the possibility to conduct assays at a larger scale.

In the pig, ejaculates are clearly ejected in different fractions. First, the pre-sperm fraction is usually discarded because of its content in urine, smegma and cell debris; the SRF fraction, with a volume of 70 to 100 mL, contains 80%–90% of the sperm cells of the ejaculate; and finally, the PSRF fraction, with a volume of 150–200 mL, which contains less than 20% of all sperm cells [11, 18, 20]. Noticeably, particular attention has been paid to the first 10 mL of the SRF fraction (SRF-P1), whose sperm have been reported to be of better quality than those contained in the rest of the SRF (SRF-P2), the PSRF and even the entire ejaculate [11, 21–24]. These differences could result from variations in total antioxidant capacity between fractions, which is higher in the SRF-P1 than in the other fractions [21].

At present, it remains unknown whether pig sperm from the separate fractions differ in chromatin protamination, chromatin condensation, and the incidence of double-stranded DNA breaks and global DNA damage. This study sought to address whether ejaculate fractions, in addition to their previously known variations in terms of sperm quality and total antioxidant capacity, also diverge in chromatin condensation and protamination, and DNA integrity.

## Methods

### Reagents

Unless specifically stated, all reagents used in the present work were purchased from Merck (Rahway, NJ, USA).

### Ethical statement

All procedures that involved animals were conducted following the European guidelines about the protection of animals used for research purposes (Directive 2010-63-EU). Semen samples were provided by a commercial artificial insemination (AI)-center (AIM Ibérica, Topigs Norsvin Spain SLU; Calasparra, Murcia, Spain), that fulfilled with European (ES13RS04P, July 2012) and Spanish (ES300130640127, August 2006) regulations and rules concerning commercialization of seminal AI-doses, and animal welfare and health.

### Boars, semen samples and sample processing

Semen donors were 8 healthy, fertile and mature (1.5 to 3 years old) boars of different breeds (Large-White and Pietrain), subjected to a routine ejaculate collection of twice a week. Boars were housed in individual pens within a controlled environment building (temperature: 15–25 °C; light: 16 h/d), with ad libitum access to water, and fed according to the nutritional requirements of AI-boars. One ejaculate per boar (*n* = 8) was collected in three separate fractions using the gloved-hand method: (i) the first 10 mL of the SRF (SRF-P1), (ii) the rest of the SRF (SRF-P2) and (iii) the post sperm-rich fraction (PSRF). Proportional aliquots of each fraction were mixed to reconstitute the entire ejaculate, which was used for the assessment of sperm quality/quantity parameters to ensure that they fulfilled the standards of commercial artificial insemination doses (> 200 × 10^6^ sperm/mL, > 70% motile sperm and < 25% sperm with abnormal morphology; as evaluated routinely in the farm with a computer-assisted sperm analysis system [ISASV1®, Proiser R + D S.L.; Paterna, Valencia, Spain] and an automated cell counter [NucleoCounter® NC-100TM; ChemoMetec, Allerod, Denmark]). While the separate fractions of the eight pig ejaculates were used for Exp. 1, the second experiment (Exp. 2) was carried out using 6 pools prepared as described below.

### Experimental design

#### Exp. 1: Analysis of chromatin protamination, condensation and DNA integrity in different ejaculate fractions

An aliquot of each ejaculate fraction (containing sperm and SP) per boar (*n* = 8) was taken to evaluate sperm chromatin protamination (Chromomycin A3 test, CMA_3_), chromatin condensation (Dibromobimane test, DBB), global DNA damage (alkaline Comet assay) and double-stranded DNA breaks (neutral Comet assay).

#### Exp. 2: Evaluation of oxidative stress index of each fraction

In order to understand better the results obtained in Exp. 1, a second experiment determining the oxidative stress index (oxidant activity divided by antioxidant capacity; seminal plasma) and sperm concentration in each fraction was devised. Sperm concentration was evaluated employing a Makler chamber (Sefi-Medical Instruments, Haifa, Israel), total antioxidant capacity of SP was determined through the cupric reducing antioxidant capacity (CUPRAC) method, and oxidant activity was assessed following the procedure described by Witko-Sarsat et al. [25]. For all these determinations, 6 pools of four animals from Exp. 1 (Example: Pool 1: Boar 1, 3, 4, 5; Pool 2: Boar 2, 4, 6, 8; Pool 3: Boar 3, 5, 7, 8; …) were used instead of individual pig samples, to remove the male effect on the volume of each fraction. These pools were formed by mixing sperm samples from animals involved in Exp. 1 for each fraction (SRF-P1, SRF-P2 and PSRF), so that 6 pools for SRF-P1, 6 pools for SRF-P2 and 6 pools for PSRF were prepared.

### Chromatin analysis

#### Analysis of chromatin protamination

Protamination of sperm chromatin was assessed with CMA_3_, an antibiotic that binds the minor DNA groove in the presence of Mg^2+^. Once bound to DNA and upon excitation at 430 nm, CMA_3_ emits fluorescence with a peak at 590 nm. Because of the organization of sperm chromatin, CMA_3_ only binds those DNA regions not associated to protamines, so that the greater the fluorescence intensity of CMA_3_, the poorer the protamination. Briefly, a stock solution at 5 mg/mL CMA_3_ in ethanol was prepared before labeling. Sperm concentration was adjusted to 20 × 10^6^ sperm/mL in PBS, samples were then diluted (1:1; v:v) in 2 × McIlvine buffer (60 mmol/L citric acid, 280 mmol/L Na_2_HPO_4_ and 20 mmol/L MgCl_2_) containing 12.5 μg/mL CMA_3_, and incubated at room temperature for 30 min. Thereafter, samples were diluted 1:10 (v:v) in filtered PBS, and then analyzed with a flow cytometer, following the protocol described below. For every sample, a negative control without CMA_3_ was included to establish basal fluorescence values.

#### Analysis of chromatin condensation

Sperm chromatin condensation was evaluated with DBB, which determines the oxidation–reduction status of disulfide bonds (R-S–S-R) and thiol groups (R-SH HS-R) of proteins. Dibromobimane is a cell-permeant, thiol-specific fluorogenic reagent that alkylates adjacent thiol pairs, located at less than 6 Å [26, 27]. Upon excitation at 394 nm and alkylation of a reduced thiol pair (R-SH HS-R) by DBB, the compound emits fluorescence at 490 nm. Increased fluorescence is thus observed when disulfide bridges between cysteine residues of protamines are not formed because the thiol groups are in a reduced state (R-SH), so that the higher the fluorescence of DBB, the lower the degree of chromatin condensation [5]. With regard to the protocol, a stock solution of DBB was first prepared by diluting 4 mmol/L DBB in 100% dimethyl sulfoxide. After adjusting sperm concentration to 1 × 10^6^ sperm/mL in PBS, samples were incubated in 20 μmol/L DBB at room temperature for 20 min. Samples were analyzed with a flow cytometer as described below. For every sample, a negative control without DBB was included in order to establish the basal fluorescence intensity of each sample.

#### Flow cytometry

Samples stained with CMA_3_ and DBB were evaluated with a flow cytometer (CytoFlex, Beckman Coulter; Fullerton, CA, USA). This device was equipped with three lasers (405, 488 and 637 nm), and fluorescence gain was calibrated daily using CytoFlex Daily QC Fluorospheres (Beckman Coulter, Fullerton, CA, USA). Dot plots were analyzed through the CytoExpert Software (Beckman Coulter, Fullerton, CA, USA), and the mean fluorescence intensity peak (arbitrary units) of the corresponding channel was exported to a .csv file that was later opened in Microsoft Excel (Microsoft, Redmond, WA, USA). For each sample, at least 10,000 sperm were evaluated at a flow rate between 10 µL/s and 60 µL/s.

Forward scatter and side scatter were used to gate the sperm population, which depicted a characteristic flame shape. For CMA_3_, samples were excited with the violet laser (405 nm), and the fluorescence emitted was collected through the Violet 610 channel (610/20 band pass). In the case of DBB, samples were excited with the violet laser (405 nm) and the fluorescence emitted was collected through the KO525 channel (525/40 band pass).

### Analysis of sperm DNA integrity

Sperm DNA integrity was evaluated as the incidence of double-stranded DNA breaks in sperm, and of global DNA damage (i.e., single- plus double-stranded DNA breaks). The alkaline and neutral variants of Single-Cell Gel Electrophoresis (Comet assay) were used to evaluate the incidence of global DNA damage and of double-stranded DNA breaks (DSB) in sperm, respectively. For this purpose, the protocol of Ribas-Maynou et al. [28], which was previously adjusted to pig sperm chromatin, was followed. Slides were treated in horizontal position through the following steps: first, sample preparation and lysis; second, electrophoresis and fixation; and finally, staining, imaging and analysis.

#### Sample preparation and lysis

First, sperm were mixed with previously melted low melting point agarose to a final concentration of 3 × 10^5^ sperm/mL and a final agarose concentration of 0.66%. Then, 6.5 mL of this mixture was placed onto an agarose pre-treated slide and then covered with an 8-mm diameter round coverslip. Two slides, one for the alkaline Comet and the other for the neutral Comet, were prepared. Samples were subsequently placed onto a metal plate kept at 4 °C for 5 min, to solidify agarose. Thereafter, coverslips were removed and slides were immersed in three lysis solutions at room temperature and incubated as follows: i) solution 1 (0.8 mol/L Tris–HCl, 0.8 mol/L DTT and 1% SDS; pH = 7.5) for 30 min; ii) solution 2 (0.4 mol/L Tris–HCl, 0.4 mol/L DTT, 50 mmol/L EDTA, 2 mol/L NaCl and 1% Tween20; pH = 7.5) for 30 min; and iii) solution 3 (0.4 mol/L Tris–HCl, 0.4 mol/L DTT, 50 mmol/L EDTA, 2 mol/L NaCl, 1% Tween20 and 100 mg/mL Proteinase K; pH = 7.5) for 3 h. Following this, slides were washed in distilled water for 2 min.

#### Electrophoresis and dehydration

Alkaline and neutral Comet slides were treated differentially when subjected to electrophoresis. Slides intended for alkaline Comet were first denatured in a cold alkaline solution (4 °C) containing 0.03 mol/L NaOH and 1 mol/L NaCl for 5 min, and then electrophoresed in an alkaline buffer (0.03 mol/L NaOH; pH = 13) at 1 V/cm for 4 min. Neutral Comet slides were directly electrophoresed in TBE buffer (0.445 mol/L Tris–HCl, 0.445 mol/L boric acid and 0.01 mol/L EDTA; pH = 8) at 1 V/cm for 12.5 min, and then washed in 0.9% NaCl solution for 2 min. After electrophoresis, slides intended for the two Comet assays were neutralized in 0.4 mol/L Tris–HCl (pH = 7.5) for 5 min, and dehydrated in 70%, 90% and 100% ethanol for 2 min each. Slides were ultimately dried at room temperature.

#### Staining, imaging and analysis

Slides were stained by immersion in 1 × SYTOX orange (Invitrogen, Whaltham, MA, USA) at room temperature for 15 min. Next, slides were washed in distilled water for 2 min, and then allowed to dry horizontally. Samples were observed under a Zeiss epifluorescence microscope (Zeiss Imager Z1, Carl Zeiss AG; Oberkochen, Germany) at 100× magnification. Comets were imaged through an AxioCam camera, coupled to Axiovision 4.6 software (Carl Zeiss; Oberkochen, Germany), adjusting exposure time to avoid overexposure of Comet heads or tails.

Image analysis was conducted using CometScore v2.0 software (RexHoover). For each image, the background was adjusted to visualize individual Comets. Although all comets were captured automatically once the background was set, a manual review of each image was further required to delete overlapping comets and debris particles and to correct the identification of Comet heads or tails, if necessary. One hundred comets per sample were captured to quantify sperm DNA integrity/damage; when this figure was not reached after manual revision, more Comets were captured. The Olive Tail Moment (OTM) was calculated for all samples, because this is the best parameter to identify the incidence of DNA breaks in a cell [29, 30]. The OTM was worked out by using the following formula: (comet tail mean intensity – comet head mean intensity) × (Comet tail intensity/Comet intensity).

### Evaluation of oxidant and antioxidant capacity of seminal plasma

In order to obtain the SP, ejaculates were centrifuged twice (1,500 × *g* at room temperature for 10 min; Rotofix 32 A; Hettich Centrifuge UK, Newport Pagnell, Buckinghamshire, England, UK) immediately after semen collection. The SP aliquots intended to oxidative stress analysis were stored in cryotubes at −80 °C until preparation of pools and further analysis. All evaluations were performed in an automated analyzer (Olympus AU400 Automatic Chemistry Analyzer; Olympus Europe GmbH, Hamburg, Germany).

Total antioxidant capacity in pooled SP samples was determined through the CUPRAC assay, which uses bathocuproinedisulfonic acid disodium salt as a chelating agent, following the protocol described by Campos et al. [31] and adapted for pig SP [12]. In brief, each SP sample (5 µL) was mixed with 0.25 mmol/L bathocuproinedisulfonic acid disodium salt (195 µL) before measuring absorbance at 480 nm. Then, 0.5 mmol/L CuSO_4_ (50 μL) was added, and the mixture was incubated at 37 °C for 4 min and 40 s. After this incubation period, the absorbance was again measured at 480 nm. The difference between the two absorbance measurements provided total antioxidant capacity. A Trolox solution in the range of 0.1–2.0 mmol/L was used to calibrate the assay, and results were expressed as mmol/L of Trolox equivalents. Each measurement was performed per duplicate in each sample. Intra- and inter-assay coefficient variations were lower than 10%, showing high linearity in serial dilutions.

Oxidant activity in pooled SP samples was evaluated through the advanced oxidation protein products (AOPPs), which are markers of oxidant-mediated protein damage, measured following the procedure described by Witko-Sarsat et al. [25] and adapted for pig SP [32]. Each sample (10 µL) was mixed with 0.074 mol/L potassium iodide (160 µL) and 50% acetic acid (25 µL). The mixture was incubated at 37 °C for 40 s, before measuring absorbance at 340 nm. Chloramine-T in the range of 0–500 µmol/L was utilized to calibrate the assay and results were expressed as μmol/L of chloramine-T equivalents. Each measurement was performed per duplicate in each sample. Intra- and inter-assay coefficient variations were lower than 10%, showing high linearity in serial dilutions.

Finally, the oxidative stress index (OSi) in each sample was calculated by considering the relative amount of oxidant products, which was assessed through the AOPPs method, and the relative total antioxidant capacity, which was determined with the CUPRAC method. The formula used was: OSi (µmol oxidants/µmol antioxidants) = AOPPs/CUPRAC (arbitrary units).

### Statistical analysis

Statistical analyses were conducted through IBM SPSS for Windows ver. 27 (IBM Corp., Armonk, NY, USA) and graphics were drawn with GraphPad Prism ver. 8 (GraphPad Software, La Jolla, CA, USA). Initially, fitting of samples with parametric assumptions (normal distribution and homogeneity of variances) was assessed through Shapiro–Wilk and Levene Tests. As no data—even after linear transformation—complied with these assumptions, non-parametric tests were run. Differences between fractions were evaluated with the Kruskal–Wallis test, and pair-wise comparisons were conducted with the Mann–Whitney test. Correlations were assessed using the Spearman test. The levels of significance was set at *P* ≤ 0.05.

## Results

### Sperm chromatin protamination is similar between ejaculate fractions

**Fig. 1:**
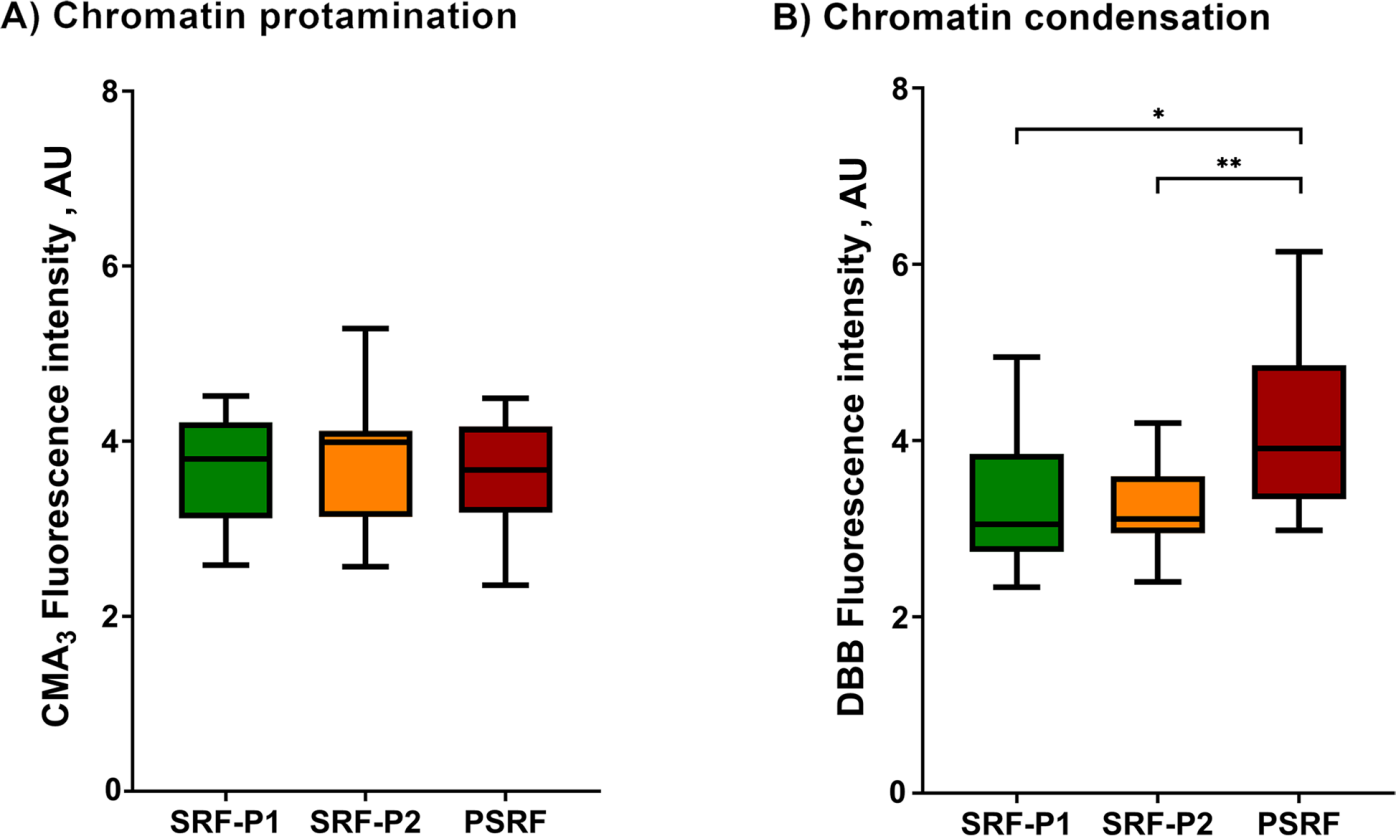
Box-whisker plot showing the effects of incubating ejaculate fractions with their respective SP on sperm chromatin protamination (**A**) and sperm chromatin condensation (**B**). The boxes enclose the 25^th^ and 75^th^ percentiles, the whiskers extend to the minimum and maximum values, and the line indicates the median. Outlier values are represented with a dot. * Indicates statistically significant differences with a *P* ≤ 0.05. ** Indicates statistically significant differences with a *P* ≤ 0.01

The protamination degree of sperm chromatin, measured inversely by CMA_3_ fluorescence intensity, was evaluated in all fractions and is shown in Fig. 1A and Additional file 1. The degree of chromatin protamination showed no significant differences between fractions (*P* > 0.05).

### Sperm chromatin is more condensed in SRF-P1 and SRF-P2 than in the PSRF

As mentioned in Methods, chromatin condensation was evaluated as the reduced status of free thiol groups (R-SH) with DBB, so that the greater the fluorescence of DBB, the lower the degree of chromatin condensation. Chromatin condensation (DBB fluorescence intensity) exhibited by the sperm from SRP-P1, SRP-P2 and PSRF is depicted in Fig. 1B and  Additional file 1. Sperm chromatin was observed to be less condensed in the PSRF than in SRF-P1 (*P* = 0.018) and SRF-P2 (*P* = 0.004).

### The incidence of DNA damage is similar between ejaculate fractions

**Fig. 2:**
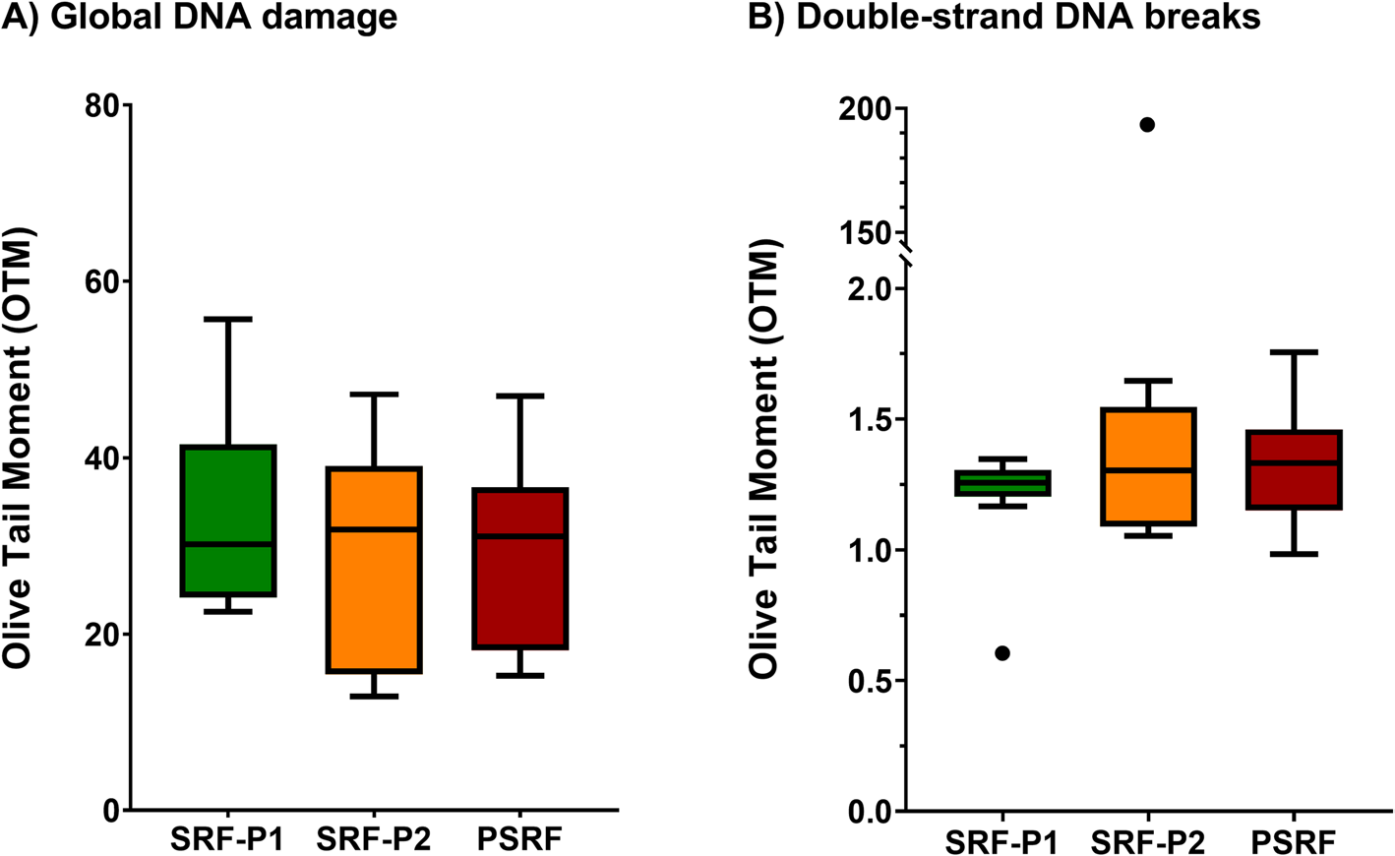
Box-whisker plot showing the effects of incubating ejaculate fractions with their respective SP on global DNA damage (alkaline Comet) (**A**), and double-stranded DNA breaks (neutral Comet) (**B**). The boxes enclose the 25^th^ and 75^th^ percentiles, the whiskers extend to the minimum and maximum values, and the line indicates the median. Outlier values are represented with a dot. * Indicates statistically significant differences with a *P* ≤ 0.05. ** Indicates statistically significant differences with a *P* ≤ 0.01

The incidence of global DNA damage, which includes both single and double-stranded DNA breaks, was evaluated in all ejaculate fractions using the alkaline Comet assay, and results are presented in Fig. 2A and  Additional file 1. Data showed that there were no significant differences between fractions (*P* > 0.05).

The incidence of double-stranded DNA breaks in sperm from all ejaculate fractions was also determined using the neutral Comet assay (Fig. 2B and  Additional file 1). Again, no significant differences between fractions were observed (*P* > 0.05).

### The incidence of global DNA damage is correlated with chromatin protamination and condensation

As shown in Table 1, whether the incidences of global and double-stranded DNA damage were correlated with chromatin protamination and condensation was also interrogated. Whereas the incidence of global DNA damage was found to be negatively correlated with both chromatin protamination (Rs = −0.688; *P* = 0.002) and condensation (Rs = −0.515; *P* = 0.029), neither protamination nor condensation were observed to be correlated with the incidence of double-stranded DNA breaks (*P* > 0.05).

**Table 1:** Correlation coefficients and the corresponding *P*-values between sperm chromatin protamination and condensation, and DNA damage (both global and double-strand DNA damage)

Parameter	Correlation coefficient (95% C.I.)	*P-*value
Sperm Chromatin Protamination		
Global DNA damage	−0.688 (−0.878 to −0.313)	0.002^*^
Double-strand DNA damage	0.377 (−124 to 0.725)	0.123
Sperm Chromatin Condensation		
Global DNA damage	−0.515 (−0.797 to −0.048)	0.029^*^
Double-strand DNA damage	0.049 (−0.440 to 0.515)	0.848

^*^ Means *P* < 0.05

### The oxidative stress index (OSi) is higher in the SRF-P1 than in SRF-P2 and PSRF, and is correlated to sperm concentration

**Fig. 3:**
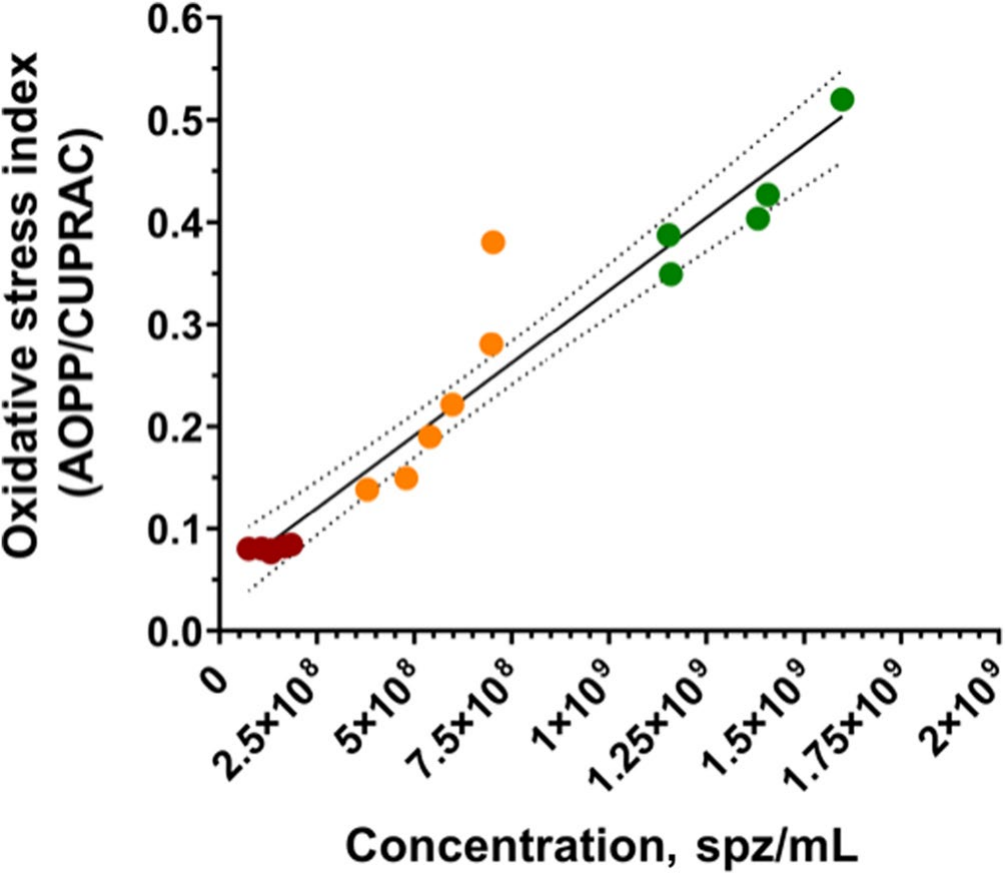
Correlations between sperm count (sperm/mL) and oxidative stress index in each ejaculate fraction (SRF-P1, SRF-P2 and PSRF). Each color represents sperm from a different fraction: red represents sperm from the PSRF, orange represents sperm from the SRF-P2, and green represents sperm from the SRF-P1

Sperm concentration and OSi, which considers oxidant activity and antioxidant capacity, of all fractions are shown in Table 2. Oxidant activity was higher in the SRF-P1 than in SRF-P2 and PSRF (*P* = 0.001 and *P* = 0.001, respectively), and antioxidant capacity was lower in the SRF-P1 than in the PSRF (*P* = 0.002). In contrast, oxidant activity was significantly higher in the PSRF than in SRF-P1 and SRF-P2 (*P* = 0.001 and *P* = 0.002, respectively), and antioxidant capacity was significantly lower in the PSRF than in the SRF-P1 (*P* = 0.002). More importantly, the relationship between oxidant and antioxidant components, evaluated as OSi, was significantly higher in the SRF-P1 than in the SRF-P2 (*P* = 0.002), and higher in the SRF-P2 than in the PSRF (*P* = 0.023) (Table 2). Furthermore, sperm concentration was found to be positively correlated to OSi (Rs = 0.973; *P* < 0.001; Fig. 3), so that it followed the same trend as OSi, the sperm concentration and OSi being the lowest in the PSRF, and the highest in the SRF-P1.

**Table 2:** Sperm concentration, oxidant activity (AOPP), total antioxidant capacity (CUPRAC) and oxidative stress index (OSi) results from the different ejaculate-fractions

	**Concentration, spz/mL**	**AOPP, µmol/L**	**CUPRAC, µmol/L**	**OSi (AOPP/CUPRAC)**
SRF-P1	1.34 × 10^9^ ± 1.88 × 10^8a^	78.90 ± 15.60^a^	188.40 ± 15.42^a^	0.42 ± 0.06^a^
SRF-P2	5.66 × 10^8^ ± 1.27 × 10^8b^	48.25 ± 11.85^b^	224.23 ± 41.66^ab^	0.23 ± 0.09^b^
PSRF	1.32 × 10^8^ ± 3.90 × 10^7c^	20.15 ± 1.28^c^	250.32 ± 9.58^b^	0.08 ± 0.00^c^

*SRF-P1* First 10 mL of the sperm-rich fraction, *SRF-P2* Rest of sperm-rich fraction, *PSRF* Post sperm-rich fraction. A total of 6 SP-samples were used for analysis, and results ptesented as mean ± standard deviation (SD)

^a,b^Different letters mean statistical differences between ejaculate-fractions (*P* < 0.05)

## Discussion

It is well known that impaired sperm quality results in reduced fertility outcomes, as data in both farm animals and humans indicate [3, 33, 34]. This has different repercussions depending on the species, from male infertility in humans to economic losses in the livestock breeding industry. In order to improve the fertilizing capacity of semen samples and counteract a potential decline in their quality, different strategies have hitherto been developed. In infertile men, oral treatments and post-ejaculation selection appear to provide sperm with greater quality, which increases the success of in vitro fertilization and intracytoplasmic sperm injection [35, 36]. In livestock, there is a selection of the best studs in farms, and only seminal doses with acceptable quality are used for artificial insemination of females [37]. In mammals, ejaculates are composed of separate fractions, and the SP of each fraction contains distinct secretions from the accessory sex glands and has a different antioxidant capacity [10]. In addition, in pigs, sperm of the SRF fraction, particularly those of the first 10-mL portion, show better morphology and motility than those of the PSRF [12, 21, 24]. Yet, whether sperm of these distinct fractions differ in the condensation and protamination of their chromatin and in the integrity of their DNA was not previously investigated.

First, the protamination degree of sperm chromatin was similar in SRF-P1, SRF-P2 and PSRF. Sperm cells condense their genetic material mostly in protamines, which replace histones during spermiogenesis and allow the DNA to be packed into toroidal structures of around 50 kb [38]. At later stages of spermiogenesis and epididymal maturation, protamine-DNA complexes are stabilized through disulfide bridges between cysteine radicals of the same protamine and of neighbor protamines [39, 40]. In both humans and farm animals, sperm protamination has been related to reproductive outcomes [41–43]. Interestingly, in a study conducted in humans, the degree of protamination of sperm chromatin was found to be positively associated to fertilization rates after intracytoplasmic sperm injection (ICSI) and to pregnancy and live birth rates [5]. These data support the use of chromatin protamination as a biomarker to predict sperm fertility. In addition, chromatin protamination and DNA integrity are known to be linked, as lower chromatin protamination increases the susceptibility of sperm DNA to be damaged [44, 45].

The evaluation of sperm chromatin condensation through DBB revealed that, whereas similar levels of disulfide bridge oxidation between SRF-P1 and SRF-P2 were observed, the degree of chromatin condensation was lower in the PSRF. The data compiled herein support that sperm of the SRF (both SRF-P1 and SRF-P2) have the most condensed chromatin, which would agree with previous observations about their higher quality and fertility potential [18, 24]. These findings are very relevant for the field of andrology, as they support that not all sperm share the same characteristics in a given sample—which was previously suggested by studies focused on sperm motile subpopulations—, but there are some cells, particularly those of the SRF-P1, that consistently present a chromatin that is more resilient to damage. Thus, and at least in pigs, chromatin condensation appears to be a marker of quality in addition to the previously reported seminal plasma biomarkers [46–49]. It is also worth bearing in mind that sperm chromatin condensation occurs during spermiogenesis and epididymal maturation, alongside other cellular processes such as changes in sperm morphology. It has been previously reported that the frequency of boar ejaculate collection affects sperm morphology and motility due to the forced transit of sperm through the epididymis, thereby having, apparently, insufficient time for epididymal maturation [50–53]. This results in a diminished sperm quality and, consequently, compromised artificial insemination outcomes [51]. Given how differences in boar collection frequency may impact sperm morphology and the relevance of spermiogenesis and epididymal maturation for chromatin condensation, it could be that a higher frequency of collection could also alter the degree of chromatin condensation observed herein for the separate ejaculate fractions. Further research on whether chromatin condensation in the different ejaculate fractions is reduced when boars are submitted to a higher collection frequency and how, if any, this has an effect on artificial insemination outcomes is, therefore, warranted.

The greater chromatin condensation observed in SRF-P1 and SRF-P2, together with the negative correlation between sperm chromatin condensation and the incidence of global DNA damage, would suggest that the sperm contained in these fractions are more resilient to DNA damage, compared to those of the PSRF. No differences between fractions (i.e., SRF-P1, SRF-P2, and PSRF), however, were found regarding DNA damage, either double-stranded DNA breaks or global DNA damage. Single and double-stranded DNA breaks are one of the major alterations of sperm chromatin. Genotoxic damage greatly impairs sperm function and may affect the integrity of DNA sequences, including those that encode for proteins. In fact, mounting evidence supports that increased sperm DNA fragmentation underlies sub/infertility [54, 55] and gives rise to embryos with impaired development and reduced implantation [56–59]. Although condensation of sperm with protamines certainly protects the DNA from genotoxic damage, agents such as reactive oxygen species (ROS) may cause DNA breaks in protaminated regions [28, 60]. Under this scenario, it is worth bearing in mind that, because sperm are almost devoid of cytoplasm, the SP contains most of the enzymatic and non-enzymatic antioxidants involved in the protection of cells from oxidative damage [12, 32]. Previous studies reported that SRF-P1 followed by SRF-P2 have the highest antioxidant capacity [21, 32], which would, in principle, not align with the similar incidence of DNA damage observed in ejaculate fractions in the present work. One could thus surmise that despite SRF-P1 and SRF-P2 having the highest amount of antioxidants, their antioxidant/oxidant balances in SP would not be favorable. In order to test this hypothesis, a second experiment (Exp. 2) was devised to determine the oxidant activity and the antioxidant capacity of SP in each of these fractions, as well as their sperm concentration. This was aimed to address whether the ROS potentially generated by the sperm contained in these fractions, especially when the number of sperm cells was high (i.e., sperm concentration), overwhelmed the antioxidant capacity of the SP, thus rendering these chemical species as potentially damaging to sperm chromatin. Results from this experiment showed a positively, significant correlation between sperm count and OSi, which indicated that the higher the sperm count, the greater the imbalance between ROS (oxidant activity) and antioxidant capacity. Thus, fractions with a higher sperm count (SRF-P1 and SRF-P2) presented greater oxidative stress despite having a higher amount of total antioxidants. High levels of ROS are known to mediate the occurrence of an increased incidence of DNA fragmentation in sperm from infertile men [61, 62]. Furthermore, a significant positive correlation between ROS and DNA fragmentation has been previously reported [63]. Yet, the present work indicates that the increased chromatin condensation exhibited by the sperm of SRF-P1 and SRF-P2 could protect their DNA from being damaged by the ROS to which they are exposed; this could explain why differences in chromatin condensation but not in the incidence of DNA damage were observed between fractions. Besides, the sperm of the PSRF showed lower chromatin condensation, which would result in an increased susceptibility to DNA damage. A greater balance between ROS levels and antioxidant capacity was, nonetheless, exhibited by the PSRF, which would mitigate the potential damaging effects of high ROS levels on sperm DNA. Taken together, all these observations could explain why no differences between ejaculate fractions were found in terms of DNA damage.

## Conclusions

In conclusion, this study demonstrated that chromatin condensation is higher in SRF-P1 and SRF-P2 compared to the PSRF. Nevertheless, similar DNA damage was observed in all ejaculate fractions, possibly as a result of the balance between oxidant activity and antioxidant capacity in each fraction. This works also supports further research evaluating how changes in the frequency of boar ejaculate collection affect the degree of sperm chromatin condensation in each fraction, and whether this has an impact on fertility outcomes.

### Supplementary Information

**Additional file 1: Supplementary Table 1.** Sperm chromatin protamination and condensation, and double-stranded and total DNA fragmentation levels exhibited by the first 10 mL of the SRF (SRF-P1), the rest portion of the sperm rich fraction (SRF-P2), and the post sperm rich fraction (PSRF).

## Data Availability

The datasets used and/or analyzed during the current study are available from the corresponding author on reasonable request.

## Abbreviations

AI: Artificial insemination
AOPP: Advanced oxidation protein products
CMA_3_: Chromomycin A3
CUPRAC: Cupric reducing antioxidant capacity
DBB: Dibromobimane
DSB: Double-stranded DNA breaks
OSi: Oxidative stress index
PSRF: Post sperm-rich fraction
SP: Seminal plasma
SRF: Sperm-rich fraction
SRF-P1: First 10 mL of the sperm-rich fraction
SRF-P2: Rest of the sperm-rich fraction
