# Supplementary material for: Chromatin condensation but not DNA integrity of pig sperm is greater in the sperm-rich fraction
Source: J Anim Sci Biotechnol. 2023 Nov 6;14:139. doi: 10.1186/s40104-023-00938-w (PMC10626759; doi:10.1186/s40104-023-00938-w)
Supplement: Supplementary file 1 — Additional file 1: Supplementary Table 1. Sperm chromatin protamination and condensation, and double-stranded and total DNA fragmentation levels exhibited by the first 10 mL of the SRF (SRF-P1), the rest portion of the sperm rich fraction (SRF-P2), and the post sperm rich fraction (PSRF). [file 40104_2023_938_MOESM1_ESM.docx]

**Supplementary Table 1** Sperm chromatin protamination and condensation, and double-stranded and total DNA fragmentation levels exhibited by the first 10 mL of the SRF (SRF-P1), the rest portion of the sperm rich fraction (SRF-P2), and the post sperm rich fraction (PSRF)

|  | **SRF-P1** | **SRF-P2** | **PSRF** |
| --- | --- | --- | --- |
| Sperm chromatin protaminization (CMA_3_ intensity), AU | 3,679.50 ± 643.63 | 3,829.58 ± 819.65 | 3,623.23 ± 672.62 |
| Sperm chromatin condensation (DBB intensity), AU | 3,308.71 ± 835.20 | 3,226.04 ± 537.89 | 4,144.33 ^*#^ ± 1025.65 |
| Double-stranded fragmentation (OTM) | 1.25 ± 0.06 | 1.32 ± 0.22 | 1.33 ± 0.24 |
| Global DNA damage (OTM) | 33.85 ± 11.28 | 28.98 ± 12.79 | 29.16 ± 11.09 |

A total of 8 samples for each ejaculate fraction were used for analysis, and results presented as mean ± standard deviation (SD)

^*^Statistical differences compared to SRF-P1 (*P* < 0.05)

^#^Statistical differences compared to the rest of SRF-P2 (*P* < 0.05)
